# Qihuang needle therapy in senile cervical spondylotic radiculopathy

**DOI:** 10.3389/fnagi.2023.1140531

**Published:** 2023-04-11

**Authors:** Juan Yang, Hongfang Huang, Jinshan Shi, Cheng He, Kun Zhang, Zhenhu Chen, Rui Zeng, Jia Liu

**Affiliations:** ^1^Traditional Chinese Medicine Rehabilitation Center, Guangzhou Dongsheng Hospital, Guangzhou, Guangdong, China; ^2^First Clinical School of Medicine, Guangzhou University of Chinese Medicine, Guangzhou, Guangdong, China; ^3^Third Affiliated Hospital of Sun Yat-sen University, Guangzhou, Guangdong, China; ^4^The First Affiliated Hospital of Guangzhou University of Chinese Medicine, Guangzhou, China; ^5^Guangzhou Emergency Medical Command Center Guangzhou, Guangzhou, China; ^6^Guangdong Food and Drug Vocational College School of Chinese Medicine Guangzhou, Guangzhou, China

**Keywords:** Qihuang needle, cervical spondylotic radiculopathy, meridian sinew, five needling, traditional Chinese medicine

## Abstract

**Background:**

Neurogenic cervical spondylosis [cervical spondylotic radiculopathy (CSR)] accounts for ~50–60% of all types of cervical spondylosis, and its incidence is the highest among all types of cervical spondylosis.

**Objective:**

The present study aimed to investigate the clinical efficacy of the Qihuang needle in the treatment of senile cervical radiculopathy.

**Methods:**

A total of 55 elderly patients with neurogenic cervical spondylosis were randomly divided into the general acupuncture group (27 cases) and the Qihuang acupuncture group (28 cases). The treatment given to these patients lasted for three sessions. The VAS scores and the Tanaka Yasuhisa Scale scores were compared before the treatment, after the first treatment, after the first session, and at the end of the session.

**Results:**

The basic data of the two groups before the treatment showed no difference. The VAS scores in the mackerel acupuncture group decreased significantly, whereas in the Tanaka Kangjiu Scale scores, the efficiency rates of the first and second courses of treatment increased significantly.

**Conclusion:**

The Qihuang needle therapy is recommended for the treatment of cervical spondylosis of the nerve root type. The said therapy is characterized by selection of fewer acupoints, a quick operation time, and no needle retention.

## Background

Neurogenic cervical spondylosis [cervical spondylotic radiculopathy (CSR)] accounts for ~50–60% of all types of cervical spondylosis, and its incidence is the highest among all types of cervical spondylosis (Wang et al., 2022). An abnormal sensation and motor disorders mainly characterize the clinical symptoms and even reduced neck, shoulders, and upper limb reflexes on account of nerve root compression. With age, the cervical disk degenerates slowly and the vertebral body bone proliferates, thus predisposing the patient to a local nerve root compression (Lin et al., 2015). With lifestyle changes, various potential predisposing factors also increase, such as the increased frequency of use of electronic products, replacement to different healthcare pillows, and improper exercise, which may induce or aggravate the disease. At present, the clinical treatment of neurogenic cervical spondylosis is mainly based on conservative treatment methods, among which acupuncture and moxibustion and tuina manipulation are commonly used clinically. Acupuncture can stretch the muscles and bones, invigorate the blood vessels, make the meridians flow smoothly, activate blood circulation, and relieve spasms and pain. The Qihuang Acupuncture Therapy uses a disposable sterile acupuncture point needle that integrates the characteristics of round needles, large needles, milli needles, and round sharp needles and serves as a characteristic therapy for treating diseases using five pricking methods. The Qihuang Acupuncture needle displays a number of features such as its hollow design that ensures the smallest needle diameter of 0.5 mm × 40 mm, 0.5 mm × 50 mm on the basis of hardness and the unique rounded shape of the needle tip, which facilitates the acquisition of Qi and effectively prevents hematoma that is caused by stabbing blood vessels. The rounded tip of the needle is slightly painful and causes little damage to the tissue. Compared with the traditional treatment, the Qihuang Acupuncture Therapy is characterized by fewer acupuncture points (only 1 to 3 acupuncture points per treatment); short operation time and a safe and easy-to-use nature (no needles are left and the operation time of individual acupuncture points is ~10 s); good efficacy and quick effect (basically, the pain can be reduced or eliminated by the needle); short treatment course (each course can be completed two to three times); and indications (the elimination of pain) and has a wide range of indications (pain diseases and nerve function of the neck are the main indicators) and other unique advantages to display new types of acupuncture treatment characteristics of Lingnan (Ling et al., 2023).

Professor Chen Zhenhu of the Department of Acupuncture and Moxibustion of the First Hospital of Guangzhou University of Traditional Chinese Medicine founded the Qihuang Acupuncture Therapy. The said therapy first improves the design of needles (Patent No. ZL2017 2 0134057.X) and takes the theory of meridians and internal organs of Chinese medicine as the basic guiding theory. Due to a lighter stimulation of acupuncture for elderly patients, fast operation, no stagnant needle retention, and avoiding the discomfort caused by the long-term fixed positioning of elderly patients, the clinical effect of Qihuang Acupuncture Therapy is good. In this study, a kind of Qihuang Acupuncture Therapy was used to treat CSR, and satisfactory clinical efficacy was achieved.

## Materials and methods

### General information

A total of 55 cases of geriatric radiculopathy patients, 24 men and 31 women, who attended the outpatient clinic of the Guangzhou Geriatric Rehabilitation Hospital from June 2021 to February 2022 were selected. They were randomly divided into the Qihuang acupuncture group (28 cases) and the general acupuncture group (27 cases) according to the random number table method. There were no differences in gender ratio, age, and disease duration between the two groups (*P* > 0.05) (Table 1). The ethics committee of the hospital approved this study. All patients signed the informed consent form.

**Table 1:** General information.

**Group**	**No. of cases**	**Gender (men/women)**	**Age (years)**	**The course of the disease (months)**
Qihuang needle	28	11/17	65.24 ± 3.31	5.1 ± 1.3

### Inclusion and exclusion criteria

The inclusion criteria for the present study were as follows: (1) those who met the relevant diagnostic criteria in the “Expert Consensus on the Standardization of Diagnosis and Treatment of Cervical Spondylosis of the Nerve” (Zhuang et al., 2015) include: (a) symptoms and signs of nerve damage caused by a localized nerve root compression; (b) the positive brachial plexus pull test or the intervertebral foramen compression test; and (c) imaging examination: X-ray and CT examinations indicate foraminal stenosis, bone hyperplasia, or osteophyte formation, and MRI suggests nerve root compression; (2) age ranging between 60 and 80 years; and (3) patients who had not received any other related external treatment before 2 weeks. The exclusion criteria for the present study were as follows: (1) patients with upper limb pain and numbness caused by other diseases, such as frozen shoulder, thoracic outlet syndrome, and tennis elbow; (2) patients with upper limb pain and numbness caused by cervical vertebral parenchymal lesions (such as tuberculosis and tumor) and skeletal deformities, (3) patients who received cervical spine acupuncture treatment or took related pain medication within 2 weeks, and (4) patients with serious medical, infectious, and mental illnesses.

### Treatment methods

#### Control group

The patients were treated with ordinary acupuncture, and the acupuncture points were selected according to the “Tenth Five-Year Plan” National Planning Textbooks for ordinary higher education. Acupoint selection: GB20, Neckjiaji, BL10, GB21, SI3, LI4, and TE5. Needle size: 0.30^*^50-mm disposable sterile acupuncture needles (Suzhou Wuzhong District Dongfang Acupuncture Instrument Factory, Batch No. 20210701). Operation procedure: The patient was asked to lie prone with a soft pillow on the chest and the hands were folded in front of the forehead to fully expose the skin of the neck and neck. After the aforementioned needle points were sterilized, the needles were inserted according to the conventional acupuncture method (attention paid to the angle and depth of needle insertion at Fengchi Point). After the needle pricking sensation was experienced by the patient, the needles were retained for 30 min and the said procedure was performed once in every 10 min. The course of treatment: 5 consecutive days of treatment is a course of treatment. After a two-day rest after each course, the second course of treatment was continued for a 3-course treatment (3 weeks in total).

####  Treatment group

The patients were subjected to Qihuang needle therapy, and the acupoints were selected. The first set of acupoints was C6 Jiaji point, IL5; the second set was C4 Jiaji point, LI14; and the third set was the extra point-Jian qian, TE4 (affected side). Needle size: 0.5^*^40-mm disposable sterile acupoint needle (Chongqing Baixiao Medical Equipment Co., Ltd., Batch No. 202110103). Operation procedure: The patient was placed in a prone position with a soft pillow on the chest and the hands were folded in front of the forehead to fully expose the skin of the neck and neck. The operator disinfected both the hands and the skin of the treatment site, the left thumb was positioned on the point, the index and the middle fingers were placed on both sides of the point, the Qihuang needle was held, and the needle was flown (inserted) into the skin quickly. After insertion, the thumb and forefinger were used to push the needle body slowly. The depth of the needle was ~25–35 mm according to the patient's body weight. First, the infusion method, with the needle tip straight to the bone surface, was used, and the needle handle was gently shaken in small increments for ~5 s. Then, the needle handle was gently swung at an angle of 15° along the longitudinal axis of the body to perform Hegu thorn needling. The needle was then fed forward and backward or left and right at an angle of 15° to 20° from the original direction, with a light and small swing of the needle handle for ~10 s. The operation time of each point was ~15 s. After the local soreness and swelling became prominent, the needle was quickly removed, and the sterile dry cotton ball was pressed for a few moments. The course of treatment: 1 day off after the first treatment, a second treatment on the third day, then a 2-day break, and a third treatment on the sixth day. Then, a day of rest was taken when a course of treatment was completed. The aforementioned three sets of acupoints were used alternately, and 3 weeks of treatment constituted a course of treatment. The same clinician does acupuncture for all patients.

### Observation of indicators and efficacy evaluation

(1) The VAS score (Park et al., 2021): A 10-cm sliding ruler was used, with the “0” end representing no pain and the “10” end representing severe pain. The patients were asked to mark the corresponding positions before and after treatment according to their pain levels. The larger the number, the more severe the pain. (2) Tanaka Yasuhisa Scale score (Huang et al., 2020): According to the patient's main complaints, symptoms, signs, hand function, and work and life ability, the higher the score, the lighter the disease. (2) Evaluation of curative effect: It was formulated with reference to “Criteria for Diagnosis and Efficacy of TCM Diseases and Syndrome” (State Administration of Traditional Chinese Medicine, 1995). Cured: Symptoms disappeared, muscle strength was , the neck and the limbs returned to normal, and the patient can participate in normal labor and work; improved: symptoms were relieved, and neck, shoulder, and back pain was relieved, and neck and limb functions improved; unhealed: symptoms did not improve.

### Statistical methods

SPSS22.0 software was used for data statistics. Variance analysis, t-test, the chi-square (χ^2^) test, and the rank-sum tests were performed for comparisons between groups. An unpaired *t*-test was used for comparison between groups and a paired *t*-test was used for intra-group pre–post comparisons. Single-ordered rank information was gathered by applying the chi-square (χ^2^) tests. A rank-sum test was used for collecting rank information. A value of *P* < 0.05 suggests that the difference was statistically significant.

## Results

### VAS scores before and after treatment

The Visual Analog Scale (VAS) scores showed no significant difference between the two groups before treatment (*P* > 0.05). Compared with the ordinary acupuncture group, the VAS scores in the Qihuang needle group decreased significantly after the first treatment, after the first session, and at the end of all sessions (*P* < 0.05). It showed that the treatment using a Qi Huang needle could effectively improve the VAS scores of patients and has a certain therapeutic effect (Table 2).

**Table 2:** Visual analog scale (VAS) score before and after treatment (mean ± SD).

**Group**	**Before treatment**	**After first treatment**	**After the first course**	**After all treatment**
Qihuang needle	6.36 ± 1.43	4.05 ± 1.21	2.89 ± 0.45	1.03 ± 0.21^*^
Ordinary acupuncture	6.42 ± 1.62	6.02 ±1.33	5.38 ± 1.27	3.52 ± 2.73^*#^
t value	0.1458	5.7497	2.0551	2.0555
P value	0.8847	< 0.001	< 0.05	< 0.05

* P < 0.05, vs. before treatment;

# P < 0.05, vs. control group after treatment.

### Tanaka Yasuhisa scale scores before and after treatment

Before treatment, the Tanaka Yasuhisa Scale scores of the two groups showed no significant difference (*P* > 0.05). Compared with the general acupuncture group, the VAS scores in the mackerel acupuncture group increased significantly after the first treatment, after the first session, and at the end of all sessions (*P* < 0.05). It was shown that Tanaka Yasuhisa Scale scores could be effectively improved in patients with Tanaka Yasuhisa Scale scores and showed a certain therapeutic effect (Table 3).

**Table 3:** Tanaka Yasuhisa Scale scores before and after treatment (mean ± SD).

**Group**	**Before treatment**	**After first treatment**	**After the first course**	**After all treatment**
Qihuang needle	8.47 ± 2.16	10.56 ± 2.63	12.83 ± 1.37	15.24 ± 1.06
Ordinary acupuncture	7.92 ± 2.42	8.74 ± 1.51	10.12 ± 1.45	11.46 ± 2.47
t value	0.8096	2.0528	7.1267	2.0550
*P*-value	0.4218	<0.05	<0.001	<0.05

### Treatment courses of two groups of patients

**Figure 1:**
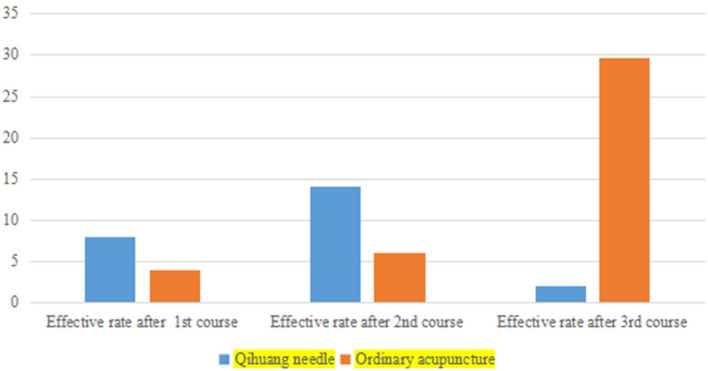
A bar chart of treatment courses between the two groups.

**Figure 2:**
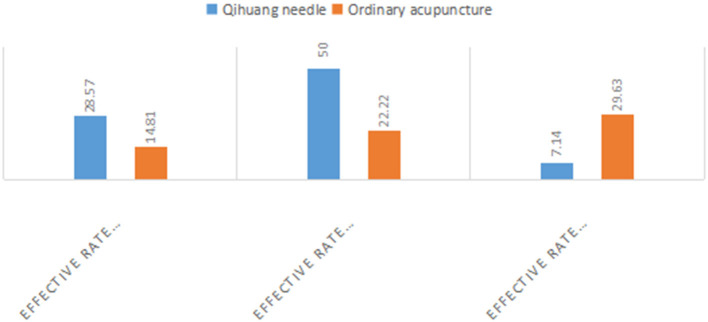
A bar chart of the ratio of treatment courses between the two groups.

The effectiveness rates after one course of treatment were 28.57% (8/28) and 14.81% (4/27) for the Qihuang acupuncture group and the general acupuncture group, respectively, and 50.00% (14/28) and 22.22% (6/27) after two courses of treatment, respectively. The effectiveness rates of the first and second courses of treatment in the Qihuang acupuncture group were higher than those in the general acupuncture group. The duration of treatment was significantly shorter in the Qihuang acupuncture group than in the control group (*P* < 0.05) (Table 4 and Figures 1, 2) (Efficacy rate = the number of patients treated effectively / total number of patients). It has been shown that the treatment effect of Qihuang acupuncture is better than that of an ordinary treatment and can shorten the course of the disease.

**Table 4:** Treatment courses between the two groups.

**Group**	**Effective rate after 1st course (cases)**	**Effective rate after 2nd course (cases)**	**Effective rate after 3rd course (cases)**
Qihuang needle	8 (28.57%)	14 (50.00%)	2 (7.14%)^①^
Ordinary acupuncture	4 (14.81%)	6 (22.22%)	8 (29.63%)

Two-group comparison, z = 2.033, P = 0.042,

① P < 0.05.

### Clinical efficacy of the two groups

**Figure 3:**
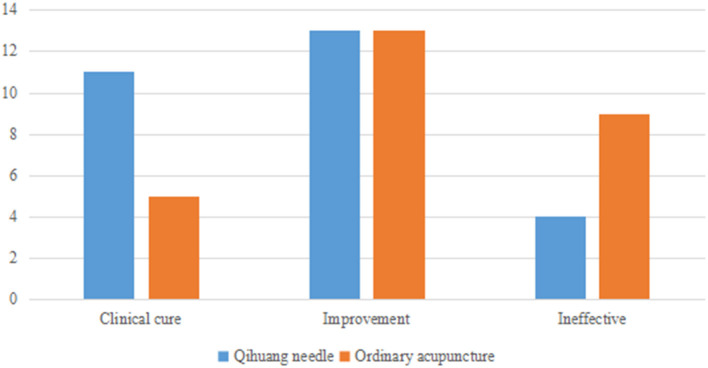
A bar chart of statistics of clinical efficacy in two groups.

**Figure 4:**
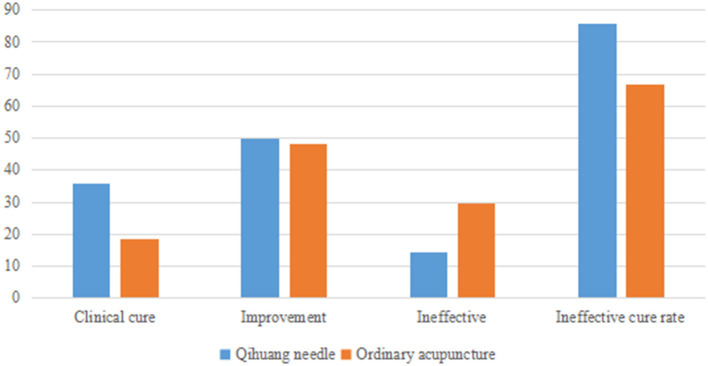
A bar chart of the composition ratio of clinical efficacy in two groups.

Cure rates were 85.71% (24/28) and 66.66% (18/27) in the Qihuang acupuncture and moxibustion and general acupuncture groups, respectively. Therefore, the clinical efficacy of the acupuncture group was better than that of the general acupuncture group (Table 5 and Figures 3, 4). It shows that the clinical treatment effectiveness of Qihuang Acupuncture Therapy is better than that of an ordinary therapy, and it is worth recommending the Qihuang Acupuncture Therapy to patients.

**Table 5:** Statistics of the composition ratio of clinical efficacy in two groups.

**Group**	**Clinical cure**	**Improvement**	**Ineffective**	**Ineffective cure rate**
Qihuang needle	11 (35.71%)	13 (50.00%)	4 (14.29%)	85.71%^①^
Ordinary acupuncture	5 (18.52%)	13 (48.15%)	9 (29.63%)	66.66%

Two-group comparison, z = 2.019, P = 0.043,

① P < 0.05.

## Discussion

Professor Chen Zhenhu pioneered the Qihuang acupuncture. The common clinical pain treatment of Qihuang acupuncture is based on the Five Body Structure Theory of the “Spiritual Book.” According to this theory, the body is composed of five bodies: skin, veins, tendons, flesh, and bone, and most of the pain symptoms originate from lesions of these five bodies. This has led to an insight into meridian-tendon differentiation-acupuncture point selection for acupuncture treatment.

“Ancesrtal sinew mainly binds bones and benefits organs.” The meridian sinew system is an external connection system, in which the Qi of the 12 meridians gathers the remaining muscles and joints. It connects the limbs and joints, constrains the bone, maintains the whole body, and governs the movement. Therefore, for the common clinical neck, shoulder, waist, and leg pain, the Qihuang needle therapy mostly uses the theory of meridian sinew as the basic guiding factor (Chen, 2020). For cervical spondylosis of the nerve root type treatment, the first step of the Qihuang needle therapy is to differentiate the meridians and tendons, i.e., according to the location of the pain distribution of the patient, so as to determine the meridian and tendon. For cervical radiculopathy, patients often experience neck pain, with radiating pain or numbness in the upper limbs as the main symptoms. Clinically, if the patient has numbness and pain in the front part of the upper limb, then it is attributed to the tendon of the Yangming meridian of the hand; but if the patient has numbness in the middle of the lateral side of the upper limb, then it is attributed to the tendon of the Shaoyang meridian of the hand. According to the identified meridians and tendons, selecting acupoints near the joints is the second step of the Qihuang acupuncture. If the pain and numbness of the patient are experienced in the upper arm, then the LI14 can be selected; but if the meridian is hand-shaoyang meridian with main numbness in the hand, then the TE4 point can be selected. At the same time, according to the symptoms of neck pain, the meridian is identified as the tendons of the musculature bladder meridian, and the neck Jiaji point adjacent to the TaiYang meridian is selected. C4 Jiaji point and C6 Jiaji point are commonly used for radiculopathy of cervical spondylosis (Wu et al., 2021). Finally, Qihuang acupuncture is derived from the five needling methods mentioned in “Lingshu Guan zhen.” The 5-point acupuncture therapy can effectively target different lesions of the body's skin–pulse–tendon–flesh–bone. For the flesh, the join valley needling is used; while for bone diseases, the transport point needling is used. However, given that patients with cervical spondylotic radiculopathy are often ill for a long time and have musculoskeletal problems, the combination of Hegu thorn needling and transport needling is often used.

Qihuang acupuncture believes that acupuncture's basis for treating diseases is the unblocking meridian–regulating Qi–harmonizing yin yang (Chen, 2022). The so-called unblocking meridian serves to open the meridians since “the meridians can determine life and death, deal with all kinds of diseases, and regulate the deficiency and excess.” The so-called regulating qi means adjusting Qi and blood, i.e., using needles and the like to adjust the Qi from “linshu ci jie zhen xie.” The so-called harmonizing yin yang is the harmony of Yin and Yang. The balance of Yin and Yang leads to a peaceful human body. In “Lingshu ben zang,” when the blood is harmonious and the meridians are unblocked, the Yin and Yang are restored, the muscles and bones are strong, and the joints are clear. To reconcile the Qi and blood of the meridians, balancing Yin and Yang has to be achieved to treat diseases.

In theory, Qihuang acupuncture still uses the TCM meridian and acupoints and acupuncture theory, but it has carried out innovative reforms in needling instruments. Professor Chen studied the characteristics of the nine needles in depth and made the needle tip of the Qihuang needle oval to “make it easier to dredge the meridian Qi between the muscles” without hurting the muscles. The needle tip has expanded the sensation of the points in depth, thus enhancing the “DeQi” effect. At the same time, the hollow structure of the needle body of the Qihuang needle increases the hardness of the needle body, which is beneficial for acupuncture manipulation of the needle handle to be transmitted to the needle tip through the needle body to achieve the effect of “Qi to the lesion.” Therefore, the design of the needle tool fully reflects the “necessity of stab and the effect of Qi.”

There are few literature reports on treating cervical spondylosis with Qihuang needle therapy, which is mainly used for treating chronic pain in the shoulder, the waist, and the knee and muscle soft tissue lesions. Zhang et al. (2020) reported that, when using a Qi Huang needle to treat low back pain diseases, it was found that some patients had a local feeling of warmth after treatment and even had the phenomenon of warm feeling being conducted. In the treatment of osteoarthritis of the knee using Qihuang Acupuncture Therapy, Wang and Chen (2017) selected the knee Yangguan, Ququan, and Guizhong points each time and treated them once in every 5 days for a total of two times. The patients' PRI, VAS, PPI scores and Lysholm knee scores were significantly lower after than those observed before the treatment, and the total effective rate was 86.67%.

Clinical studies Li Q. (2010) suggest that acupuncture at the Jiaji point can relieve muscle spasms by loosening the adhesion of local tissues, thereby improving the ischemia and hypoxia state of neck tissues to relieve local pain and nerve compression symptoms. Furthermore, researchers suggest that Jiaji acupoint combined with acupuncture can induce the body to release opioid peptide-like analgesic substances and promote the absorption of local aseptic inflammation (Liu et al., 2016). Furthermore, by performing Hegu thorn needling on the acupoints where the meridians and tendons of the upper limbs converge, the Qi and blood between the muscles can be redistributed *via* the effect of Hegu thorn (Wang, 2018). The improvement in Qihuang acupuncture has enhanced the effect of acupuncture on “DeQi” and meridian-Qi conduction. Therefore, acupuncture at Jiaji point and the acupoints where the meridians and tendons of the upper limbs gather can take effect quickly and can relieve local pain symptoms in the neck and nerve root compression, thereby displaying a good relief effect (Fu et al., 2021; Yan et al., 2021).

The Qihuang needle treatment of cervical spondylotic radiculopathy has a quick onset effect. The VAS score shows that the patients in the Qihuang needle group can improve their pain and numbness after one treatment. Compared with the ordinary needle group, the Tanaka Yasuhisa Scale showed that the Qihuang needle therapy significantly improved patients' symptoms, signs, hand function, and quality of life. The overall evaluation rates of the single course of treatment were 28.57 and 14.81% for the Qihuang acupuncture treatment and general acupuncture groups, respectively, and the Qihuang acupuncture treatment group was significantly higher than the general acupuncture group. The Qihuang acupuncture treatment for two courses of treatment was 50.00% higher than the 22.22% in the control group, suggesting that the Qihuang needle treatment was more effective than ordinary acupuncture. The clinical efficacy statistics showed that the cure rate of the Qihuang needle group was better than that of the control group.

## Conclusion

The Qihuang needle therapy is recommended to treat patients with cervical spondylosis of the nerve root type. The needles show the maximum advantages of using nine classical needles. Guided by the theory of meridians and tendons, the meridians and tendons are selected to gather at the local acupoints of the joints. The Qihuang needle therapy shows characteristics such as the selection of fewer acupoints, a quick operation time, and no needle retention. In addition, it causes less irritation and brings about a rapid clinical effect in patients with senile cervical radiculopathy, which is suitable for clinical application.

## Data availability statement

The original contributions presented in the study are included in the article/supplementary material, further inquiries can be directed to the corresponding author.

## Ethics statement

The studies involving human participants were reviewed and approved by the Hospital's Ethics Committee. The patients/participants provided their written informed consent to participate in this study. Written informed consent was obtained from the individual(s) for the publication of any potentially identifiable images or data included in this article.

## Author contributions

JY and HH were major contributors in writing the manuscript. JS and CH collected the patient data. KZ and ZC performed both surgeries and followed up the patients. RZ and JL realized the scarcity of the two cases, did literature searches, and revised the manuscript. All authors read and approved the final manuscript.
